# Can We Prevent Mitochondrial Dysfunction and Diabetic Cardiomyopathy in Type 1 Diabetes Mellitus? Pathophysiology and Treatment Options

**DOI:** 10.3390/ijms21082852

**Published:** 2020-04-19

**Authors:** Aleksandra Cieluch, Aleksandra Uruska, Dorota Zozulinska-Ziolkiewicz

**Affiliations:** Department of Internal Medicine and Diabetology, Poznan University of Medical Sciences, 60-834 Poznan, Poland; aleksandrauruska@gmail.com (A.U.); zozula@box43.pl (D.Z.-Z.)

**Keywords:** mitochondrial dysfunction, diabetic cardiomyopathy, oxidative stress

## Abstract

Type 1 diabetes mellitus is a disease involving changes to energy metabolism. Chronic hyperglycemia is a major cause of diabetes complications. Hyperglycemia induces mechanisms that generate the excessive production of reactive oxygen species, leading to the development of oxidative stress. Studies with animal models have indicated the involvement of mitochondrial dysfunction in the pathogenesis of diabetic cardiomyopathy. In the current review, we aimed to collect scientific reports linking disorders in mitochondrial functioning with the development of diabetic cardiomyopathy in type 1 diabetes mellitus. We also aimed to present therapeutic approaches counteracting the development of mitochondrial dysfunction and diabetic cardiomyopathy in type 1 diabetes mellitus.

## 1. Introduction

Type 1 diabetes mellitus (T1DM) is an organ-specific autoimmune disease caused by the selective destruction of pancreatic beta cells, leading to an absolute insulin deficiency. Disturbances of the glucose metabolism in diabetes are associated with changes in the energy metabolism, particularly during insulin deprivation [1]. The lack of endogenous insulin, together with insulin resistance, and combined with exogenous insulin delivery, makes T1DM a disease of energetic destruction.

The main organelles responsible for energetic homeostasis are mitochondria. These semi-autonomous organelles are cellular energy centers, the main function of which is the production of adenosine triphosphate (ATP), necessary for the proper functioning of cells [2]. Thanks to respiratory chain complexes located in the inner mitochondrial membrane (IMM), cellular respiration processes occur. The stages of respiration occurring in the mitochondria are the Krebs cycle and the respiratory chain [3]. As mitochondria play a fundamental role in the metabolism of oxygen, it is extremely important to look at their effects in patients with various diseases including diabetes. Scientific reports in recent years have identified quantitative, qualitative, and functional disorders within the mitochondria and have linked these phenomena to the occurrence of chronic complications of diabetes including cardiomyopathy. However, despite their important functions, mitochondria can become an enemy to the human body. During oxidation, they generate reactive oxygen species (ROS). There is evidence that ROS are clinically relevant to the pathogenesis of long-term complications of diabetes [4].

Diabetic cardiomyopathy is defined by the occurrence of abnormal myocardial structures and performance without other cardiac risk factors, such as coronary artery disease, hypertension, or significant valvular disease, in patients with diabetes mellitus. This condition begins with myocardial fibrosis, dysfunctional remodeling, and associated diastolic dysfunction. Then, there is systolic dysfunction, and finally, clinical heart failure [5]. Cardiac dysfunction applies to both T1DM and type 2 diabetes mellitus (T2DM). Regarding pathogenesis in terms of mitochondrial function, the data are contradictory. Namely, the majority of studies indicated the role of mitochondrial dysfunction in T1DM. However, Marciniak et al. indicated that both T1DM and T2DM present cardiac dysfunction. However, mitochondrial respiration impairment is only obvious in T2DM. Interestingly, T2DM mice developed cardiac alterations associated with a reduction in mitochondrial oxygen consumption, despite an increase in mitochondrial biogenesis signaling [6].

Mitochondria were investigated several decades ago when scientists discovered a reduced number of mitochondria in the heart accompanied by mitochondrial swelling and damage to the mitochondrial membranes and cristae in alloxan diabetes [7]. Most studies that aimed to evaluate mitochondrial dysfunction in T1DM were conducted on various experimental animal models (alloxan- and streptozotocin-injected animals, as well as depancreatized animals) [8]. Recent reports have revealed disorders within the mitochondria, not only in terms of their counts or morphology [9], but also their function. The current review aimed to collect scientific reports linking disorders in mitochondrial function with the development of diabetic cardiomyopathy in T1DM. The review aimed to present therapeutic approaches counteracting the development of mitochondrial dysfunction and diabetic cardiomyopathy in T1DM.

## 2. The Mitochondrial Oxidative Phosphorylation (OXPHOS) System and Respiratory States

The OXPHOS system consists of the electron transport chain (ETC), which comprises nicotinamide adenine dinucleotide hydride (NADH) dehydrogenase (complex I), succinate dehydrogenase (complex II), cytochrome c oxidoreductase (complex III), and cytochrome c oxidase (complex IV). Protein complexes are involved in electron transport. Transformations in those complexes allow the generation of a potential difference across the IMM, which causes the synthesis of ATP when protons return to the mitochondrial matrix via ATP synthase (Complex V) [10].

The animal model of T1DM showed a significant reduction in the activity of OXPHOS complexes in cardiac mitochondria (Table 1). Evaluation of the OXPHOS functionality by Ferreira et al. revealed a significant decrease in all respiratory complex activity, except complex III in the heart mitochondria of rats aged 6–8 weeks treated with a single intraperitoneal injection of streptozotocin (60 mg/kg). The effect of T1DM on animal heart mitochondria was assessed after four weeks of streptozotocin administration [11]. Abnormalities in the OXPHOS activity were accompanied by a significant increase in one of the uncoupling proteins of the IMM—uncoupling protein-3 (UCP-3) and a decrease in the mitochondrial level of cytochrome c, which physiologically transfers electrons between complexes III and IV.

The higher level of UCP-3 does not protect the heart mitochondrial membrane proteins from oxidative damage. Instead, a decrease of the mitochondrial transcriptional factor A (TFAM) content suggested impaired regulation of the mtDNA gene expression in heart mitochondria. In turn, Toccheti et al. observed a decrease in respiration, but only with the complex II and IV substrates and markedly lower adenosine diphosphate (ADP) phosphorylation rates in the mitochondria of T1DM guinea pig hearts. Animals were treated with a single intraperitoneal injection of streptozotocin (80 mg/kg) [12].

Vazquez et al. found that the mechanism for the decreased mitochondrial oxidative phosphorylation in the diabetic heart decreased NADH coenzyme Q oxidoreductase and succinate-coenzyme Q oxidoreductase of complexes I and II, respectively. They assessed the heart mitochondria of two-month-old rats treated with a single intraperitoneal injection of streptozotocin (55 mg/kg) 10 weeks after the onset of hyperglycemia. Interestingly, abnormalities in the functioning of mitochondria developed early during the evolution of T1DM, before heart failure occurred. They indicated the complex pathogenesis of mitochondrial electron transport defects in T1DM, including a decreased amount or loss of catalytic activity of ETC subunits [13].

Vadvalkar et al. proved that Akita mice heart mitochondria had significantly impaired state 3 respiration with physiological pyruvate. They observed a decrease in the rate of pyruvate transport in the animals’ heart mitochondria; however, this was not accompanied by a decrease in the mitochondrial pyruvate carriers 1 and 2. The authors suggest acetylation within mitochondrial pyruvate carrier 2 as a causative factor of disorder in the pyruvate transport activity and metabolic changes in the diabetic heart [14].

Pham et al. found that mitochondria from T1DM rat hearts treated with an injection of streptozotocin (55 mg/kg) eight weeks after the onset of hyperglycemia were less efficient than those from non-diabetic hearts and produced less ATP in normoxic steady-state respiration states. Also, these mitochondria released more net ROS per oxygen consumed in OXPHOS states. Despite the considerable suppression of the ATP synthesis capacity in oxygen-saturated states, the mitochondria from T1DM hearts appeared to consume as much ATP in anoxic infarct-like states as the mitochondria from non-diabetic hearts. The authors suggested that this could produce a greater ATP deficit on reoxygenation [15].

Bugger et al. demonstrated tissue-specific remodeling of the proteome of the mitochondrial energy metabolism in T1DM Akita mice [16]. They revealed that, in comparison to other tissues, such as liver, kidney, or brain tissue, the cardiac mitochondria were characterized by a decrease in state 3 respiration, ATP synthesis, mitochondrial cristae density, and tricarboxylic acid (TCA) cycle proteins. They found repression of the OXPHOS subunits with reduced expression of the OXPHOS subunit genes. Surprisingly, the mitochondrial function of other assessed tissues was unaffected. Additionally, the transcriptional changes were accompanied by reduced expression of peroxisome proliferator-activated receptor gamma coactivator 1-alpha (PGC-1α), peroxisome proliferator-activated receptor gamma coactivator 1-alpha (PGC-1β), estrogen-related receptor α (ERRα), TFAM, and peroxisome proliferator-activated receptor (PPARα). Thus, the authors of the study gave a new insight into the pathogenesis of remodeling the mitochondrial proteome and impaired mitochondrial function in cardiac mitochondria, indicating the participation of transcriptional mechanisms.

## 3. Oxidative Stress in T1DM Hearts

**Figure 1:**
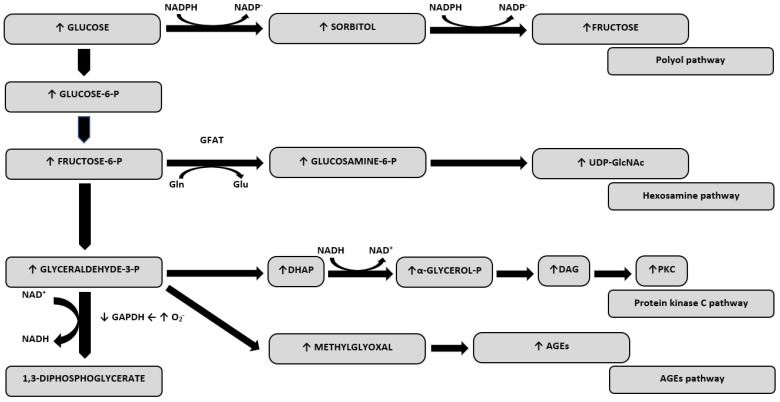
Oxidative stress-related pathways derived from hyperglycaemia. Abbreviations: GAPDH, glyceraldehyde-3-phosphate dehydrogenase; DHAP, dihydroxyacetone phosphate; DAG, diacylglycerol; PKC, protein kinase C; GlcNAc, O-linked N-acetylglucosamine; GFAT, glutamine:fructose-6-phosphate aminotransferase; Gln, glutamine; Glu, glutamate; NAD+, nicotinamide dinucleotide; UDP, uridine diphosphate.

Chronic hyperglycemia is a major cause of diabetes complications. Hyperglycemia induces mechanisms that generate the excessive production of ROS [4]. This phenomenon, together with the reduced concentration and activity of antioxidants, leads to the development of oxidative stress (Figure 1). The oxidative stress mechanisms in diabetes can be regulated not only by drugs, but also exercise [17] and epigenetic factors [18].

Tocchetti et al. showed that exposure to high glucose combined with β-adrenergic stimulation via isoprenaline resulted in higher oxidative stress in T1DM guinea pigs compared with the controls. Moreover, the mitochondria of T1DM hearts presented higher H_2_O_2_ emissions when challenged with oxidants [12]. Malondialdehyde (MDA) is a by-product of lipid peroxidation, and thus an indicator for oxidative damage. Data suggested that several protein bands in diabetic OVE26 hearts contained more MDA than nondiabetic hearts [19]. Semaming et al. showed that the plasma MDA level and mitochondrial ROS production were significantly increased in diabetic rats when compared with normal rats [9]. Makino et al. observed increased cytosolic and mitochondrial O_2_ concentrations in mouse coronary endothelial cells (MCECs) in diabetic (mice aged six weeks treated with a single injection of streptozotocin (60 mg/kg) compared with the control mice. The oxidative stress marker 8-iso-prostaglandin F2α (8-iso-PGF2α) increased in the plasma of diabetic mice. Additionally, they observed that high glucose treatment over 24 h significantly increased the cytosolic and mitochondrial O_2_^−^ concentrations in human coronary endothelial cells (HCECs) [20].

The role of calpain activity in the progression of cardiac pathologies is known [21]. Ni et al. showed elevated mitochondrial calpain-1 protein levels and activity in T1DM mouse hearts. This increase was accompanied by elevated mitochondrial ROS production, oxidative damage, and a reduction in the ATP synthase-α (ATP5A1) protein and ATP synthase activity. They suggested that calpain-1 caused the downregulation of the ATP synthase-α protein and the disruption of ATP synthase, stimulating mitochondrial ROS production and leading to diabetic cardiomyopathy. They revealed that the inhibition of mitochondrial calpain increased the protein level and ATP synthase activity, decreasing the oxidative stress and preventing diabetic hearts. The targeted upregulation of calpain-1 in mitochondria induced the cleavage of ATP5A1, superoxide generation, and apoptosis in cardiomyocytes. However, the upregulation of ATP5A1 restored the ATP synthase activity and decreased the mitochondrial ROS generation in diabetic hearts [22].

Tsai et al. showed the upregulation of Ras protein-specific guanine nucleotide releasing factor 1 (RasGRF1) expression in the heart tissue of T1DM mice. RasGRF1 mediated the inflammation and oxidative stress contributing to diabetic cardiomyopathy. Chronic diabetes upregulated the oxidative stress markers NOX2 and NOX4 in heart tissue. A deficiency of RasGRF1 attenuated these processes and improved the diastolic functions in diabetic mice [23].

## 4. Interfibrillar Mitochondria (IFM) vs. Subsarcolemmal Mitochondria (SSM) in T1DM Hearts 

Myocardial mitochondria comprise two spatially distinct subpopulations displaying different anatomy, biochemistry, and functions [24]. These subpopulations are referred to as interfibrillar mitochondria (IFM) and subsarcolemmal mitochondria (SSM). The distinction between these two subpopulations of mitochondria is intentional due to the differences in their responsiveness to hyperglycemia and metabolic stress with the enhanced susceptibility of IFM in T1DM. Thus, IFM could be the preferred target for drug treatment to prevent cardiomyopathy.

Thapa et al. [25] revealed significant decrements in the ETC complex I, III, IV, and V activities, state 3 and state 4 respiration rates, and mitochondrial membrane potential within diabetic IFM as compared with the healthy control IFM, with no significant differences between the control and diabetic SSM. They observed no significant differences in the complex I, III, IV, and V contents in SSM and IFM between the control and diabetic groups. Diabetic IFM were characterized by an increase in the lipid peroxidation by-products 4-hydroxyalkenal (4-HAE) and MDA compared to the control IFM, with no significant differences observed in the diabetic SSM when compared to the control SSM. The evaluation of IFM mitochondria showed its altered morphology with damaged cristae structures and decreased internal complexity when compared to the control heart.

Baseler et al. [26] indicated that mitochondrial proteomic alterations in T1DM were more pronounced in the IFM. They showed that diabetic IFM, compared to a control, were characterized by decreased carnitine palmitoyltransferase-1, an outer mitochondrial membrane protein that mediates the transport of long-chain fatty acids across the membrane. Additionally, these mitochondria have a diminished number of respiratory chain proteins from all respiratory complexes, decreased proteins of the citric acid cycle and other proteins necessary for energy production, and decreased proteins involved in the transport of proteins and/or substrates across the IMM. The authors found decreased mitofilin, a transmembrane IMM protein that regulates mitochondrial cristae morphology, and diminished prohibitin-2, a protein involved in mitochondrial chaperone functions, which may impact mitochondrial morphology, as well as the maintenance of mitochondrial DNA, and decreased mitochondrial heat shock protein 70 in diabetic IFM. T1DM caused a decrease in the mitochondrial membrane potential in IFM, which is essential for protein translocation across the IMM. All the above changes were unaffected by T1DM in SSM, and an increase was even observed, which can be considered a compensatory mechanism. The authors suggest that adverse phenomena focus primarily on the mitochondria located between the myofibrils and that these changes underlie the mitochondrial dysfunction in diabetic hearts.

Cardiolipin is a unique phospholipid, primarily located in the IMM, and is thought to play a critical role in mitochondrial structure and bioenergetics. The properties of cardiolipin allow it to interact with IMM proteins and to facilitate proper mitochondrial function. The literature suggests an interaction between cardiolipin and the ETC complexes involved in oxidative phosphorylation. Pathological changes in the amount of cardiolipin can have deleterious consequences for mitochondrial function and can trigger the production of reactive oxygen species [27]. Croston et al. [28] showed that T1DM negatively affected the cardiolipin biosynthetic pathway specifically at cardiolipin synthase (CRLS), which resulted in decreased cardiolipin content and the loss of interactions with key ATP synthase F0 complex constituents in the IFM. They revealed a significant decrease in the CRLS protein content in diabetic IFM, with a concomitant decrease in its activity and a decrease in the ATP synthase activity as compared to the control, with no change in the SSM. The authors suggested that the decreased cardiolipin content associated with T1DM resulted from defective cardiolipin biosynthesis, which influenced its association with IMM proteins. Thus, the preservation of CRLS and IMM is a potential therapeutic target that may be influenced to prevent cardiomyopathy associated with T1DM hearts.

The mitochondrial ATP-sensitive potassium channels (MitoK_ATP_) work in organellar volume regulation, apoptosis, and ischemic preconditioning [29]. Fancher et al. determined the effects of T1DM on the function and expression of mitoK_ATP_ in cardiac mitochondria. MitoK_ATP_ is composed of a pore-forming subunit (Kir6.1) and a diazoxide-sensitive sulphonylurea receptor—a regulatory subunit (SUR1). The authors observed that diabetes significantly reduced Kir6.1 and SUR1 expression in IFM. Similarly, diabetes significantly reduced the Kir6.1 expression in SSM. However, the SUR1 expression in SSM was unaffected. Reduced mitoKATP expression in IFM was accompanied by the suppression of its function in the form of reduced diazoxide-induced depolarization of mitochondrial membrane potential [30]. MitoK_ATP_ is another therapeutic target that could protect the heart against ischemia-reperfusion injury [31].

## 5. Protection of Mitochondria in T1DM Hearts

The chance to counteract the development of diabetic cardiomyopathy in T1DM may entail the clinical use of substances whose effectiveness have been demonstrated in animal models (Table 2).

### 5.1. Role of Insulin and Combined Therapy

Semaming et al. showed that diabetes increased oxidative damage, depressed heart rate variability, caused cardiac contractile dysfunction and decreased cardiac anti-apoptotic BCL2 protein levels. The authors proved that treatment with insulin, protocatechuic acid (PCA), or combined therapy (insulin with PCA) can reverse these unfavorable conditions in T1DM. PCA, a major metabolite of anthocyanins, has been shown to have antioxidant, antitumoral, and anti-inflammatory activities. The mentioned treatments significantly decreased the plasma MDA level and ROS production and attenuated the mitochondrial depolarization and mitochondrial swelling compared with a control. These treatments improved the heart rate variability, attenuated cardiac dysfunction, prevented cardiac mitochondrial dysfunction, and increased the anti-apoptotic BCL2 protein expression, suggesting apoptosis prevention in diabetic rats. Interestingly, combined therapy, but not insulin alone, restored the cardiac contractile dysfunction to normal status [9]. This study highlighted the role of combination therapy in the prevention of diabetic cardiomyopathy, without forgetting the significant contribution of insulin, which is the drug of choice in the treatment of T1DM. Tocchetti also noticed the role of insulin in preserving proper cardiac redox balance, however, indicating that there is another more effective substance preventing cardiac function. The authors showed that the chronic administration of insulin restores a proper cardiac redox balance, however, without rescuing the mitochondrial respiration and contractile performance, whereas acute palmitate infusion (except for the restoring of the redox balance) preserved the function of the cardiomyocytes. Thus, these treatments prevented high glucose combined with beta-adrenergic stimulation-induced cardiac dysfunction [12].

Da Silva et al. proved that, in rats with T1DM, swimming training corrected pathologies, such as reduced [Ca(^2+^)]I transience, increased uncoupling protein-2 expression, and increased Ca(^2+^) uptake in the heart mitochondria [32]. Moreover, insulin treatment further normalized the Ca^2+^ transient amplitude, nicotinamide adenine dinucleotide phosphate hydride (NADPH) oxidase-4 expression, and carbonyl protein contents in the left ventricular (LV) tissue. The combination of both therapies restored the LV tissue superoxide dismutase and mitochondrial O_2_ consumption, H_2_O_2_ release, and permeability transition pore (MPTP) openings in the heart mitochondria. The authors noted the protective effect of both physical activity and insulin therapy on oxidative stress, Ca^2+^ homeostasis disruptions, and mitochondrial dysfunctions in the hearts of rats with T1DM while emphasizing the role of combination therapy in obtaining the most optimal effect.

In the study of Remor et al., 30 days of the hyperglycemic condition provoked a significant inhibition of the activities of the mitochondrial complexes I, II, and IV in cardiac muscles, whereas a significant complex IV inhibition occurred with as few as ten days of treatment. Insulin administration was effective in protecting against the hyperglycemia-induced inhibition of mitochondrial OXPHOS enzymes, probably due to its demonstrated capacity to modulate the levels of mRNA for genes encoding the respiratory chain complexes. As insulin exerts part of its protective activity through activation of the phosphoinositide-3-kinase (PI3K)-Akt pathway, the authors suggested that the severe changes in the energy metabolism, elicited by both glucose toxicity and the lack of insulin were mediated by the inhibition of Akt signaling. Thus, early and continuous insulin therapy has an essential role in the maintenance of mitochondrial homeostasis in T1DM [33].

### 5.2. Role of Antioxidants

Catalase is an enzyme known to be protective against oxidative stress [34]. Catalase is mainly found in peroxisomes. Peroxisomes and mitochondria share a redox-sensitive relationship. Defects in catalase activity have been shown to induce mitochondrial oxidative stress in various organs. Interestingly, the increase in the mitochondrial redox state is closely associated with the induction of peroxisomal function loss [35]. Ye et al. showed the destruction of mitochondria and an increase in MDA-modified proteins in diabetic OVE26 mice cardiomyocytes compared to controls and diabetic hearts overexpressing the antioxidant protein catalase [19]. The overexpression of catalase assured significant protection from the damage induced by diabetes, which was proven by the reduction of the MDA level in diabetic hearts. Catalase overexpression protected diabetic hearts from a significant increase in ROS production caused by the exposure of OVE26 cardiomyocytes to high glucose concentrations.

The exposure to high glucose did not affect the ROS level in the control myocytes. The chronic overexpression of catalase, similar to the acute in vitro treatment with inhibitors of complex I and II (rotenone and thenoyltrifluoroacetone, respectively), eliminated the excess production of ROS in OVE26 cardiomyocytes in these conditions. The overexpression of catalase has been shown to prevent morphologic damage to mitochondria and myofibrils in T1DM mice and was observed to improve the cardiomyocyte contractility. This study demonstrated the importance of ROS participation in the development of diabetic cardiomyopathy, and that they are the source of oxidative damage in diabetic hearts.

It is possible to influence not only the function but also the morphology of the mitochondria. Makino et al. showed in mouse coronary endothelial cells (MCECs) that changes in the morphology of mitochondria, such as their fragmentation in T1DM, can be restored by the administration of 4-hydroxy-2,2,6,6-tetramethylpiperidine 1-oxyl (TEMPOL, an O_2_^−^ scavenger). The administration of the O_2_^−^ scavenger was connected with a decrease in oxidative stress (significant decrease in oxidative stress marker 8-iso-PGF-2α) in diabetic mice, and this decreased the oxidized level of several proteins in the heart. However, this procedure did not influence the protein level responsible for the mitochondrial dynamics. The optic atrophy 1 (OPA1) protein level was significantly decreased and the dynamin-related protein 1 (DRP1) level was significantly higher in MCECs freshly isolated from diabetic mice compared with control mice. The authors did not observe fusion and fission- related protein level changes during this treatment, which suggested that improvement of the mitochondrial morphology may be a direct influence from the decrease in O_2_^−^ concentration from the scavenger treatment, and not the effect of changes in these proteins levels. The O_2_^−^ scavenger treatment restored mitochondrial adverse changes induced by high glucose treatment in human coronary artery endothelial cells (HCECs), such as the decrease in mitochondrial volume. However, the scavenger treatment did not influence the increased level of DRP1 in hyperglycemic conditions in HCECs, which could contribute to intensified mitochondrial fragmentation independent of the increased O_2_^−^ production. In contrast to MCECs, the effect of high glucose treatment on mitochondrial fragmentation was not related to a change in the OPA1 protein level [20].

Mitochondria phospholipid hydroperoxide glutathione peroxidase 4 (mPHGPx) is an antioxidant enzyme capable of scavenging membrane-associated lipid peroxides in the IMM. Researches assessed whether the overexpression of mPHGPx could reverse the mitochondrial dysfunction associated with diabetic hearts in T1DM. The overexpression of mPHGPx restored state 3 and state 4 respiration rates, preserved mitochondrial respiratory chain proteins, preserved ETC complex I, III, and IV activities, enhanced ATP synthase activity, reversed mitochondrial protein import dysfunction, and attenuated H_2_O_2_ production in diabetic IFM. Moreover, mPHGPx expression decreased the accumulation of lipid peroxidation by-products, suggesting the ability of mPHGPx to attenuate ROS-induced damage to lipids within the IFM. mPHGPx overexpression is another proposal to preserve the mitochondrial proteome and provide cardioprotective benefits to diabetic hearts in T1DM [36].

The mitochondrial enzyme aldehyde dehydrogenase 2 (ALDH2) was shown to rescue against diabetic-induced cardiomyopathy injury. Guo et al. suggested that ALDH2 exerted protective effects against diabetes-induced cardiac toxicity and myocardial dysfunction through promoting AMPK-dependent autophagy [37]. They showed that diabetes or high glucose-induced alterations in cardiac mechanical and autophagic responses might be associated with a dampened and elevated phosphorylation of AMP-dependent protein kinase (AMPK) and FOXO3a, respectively. ALDH2 and its agonist Alda-1 are likely to offer protection by reversing diabetes or significant glucose-induced changes in AMPK and FOXO3a signaling, en route to improved autophagy, cardiac geometry, and mechanical function. Their observations suggested that the induction of autophagy may be a potential therapeutic strategy for hyperglycemic cardiotoxicity in T1DM.

### 5.3. Role of Mitofilin

Mitofilin is a protein found in large amounts in the heart. It occurs in IMM where it is specifically localized to the cristae junction. Mitofilin is a key component of the mitochondrial site and a central organizer of mitochondrial architecture, cristae junctions, and cristae morphology. Additionally, this protein is responsible for maintaining proper mitochondrial function [38,39]. Thapa et al. [25] showed that mitofilin overexpression in T1DM preserved ETC complex I, III, IV, and V activities, and restored state 3 respiration rates in diabetic IFM as compared to diabetic IFM. The significant reduction of mitochondrial membrane potential in the diabetic IFM was restored by mitofilin overexpression. Mitofilin overexpression decreased the accumulation of lipid peroxidation by-products in diabetic IFM, which suggests an ability of mitofilin to attenuate the ROS-induced damage to lipids. Mitofilin restored diabetes-induced damage of cristae structures in the diabetic IFM, suggesting its role in the maintenance of cristae morphology. However, mitofilin did not alter the levels of proteins involved in mitochondrial dynamics.

### 5.4. Non-Pharmacological Options of Heart Protection

A controversial observation was made by Zhang et al., who revealed that exposure to low-dose radiation (LDR) at medium or high doses (25 or 50 mGy, respectively), but not 12.5 mGy, significantly prevented cardiac apoptosis. This exposure strongly inhibited cardiac P53 activation—an upstream inducer of the mitochondrial death pathway. Additionally, the increased ratio of Bax to Bcl as a mitochondrial cell death pathway was strongly, but not completely suppressed, suggesting that LDR prevented diabetes-induced cardiac cell apoptosis partially because of the inhibition of the mitochondrial pathway. The exposure to radiation significantly decreased the contents of such classic oxidative damage markers as 3-nitrotyrosine (3-NT), 4-hydroxy-2-nonenal (4-HNE), and MDA in T1DM hearts. This exposure also strongly inhibited the ROS production in diabetic hearts, improved the Akt activation, and enhanced the nuclear factor erythroid 2-related factor 2 (Nrf2) function—an important cellular defense mechanism against oxidative stress. Thus, an ideal drug to prevent diabetes-induced cardiomyopathy may need to inhibit oxidative stress and apoptosis simultaneously, which can possibly be provided by LDR [40].

## 6. Summary

There is strong evidence that mitochondrial dysfunction plays an important role in the pathogenesis of cardiomyopathy in T1DM. The differences in the responsiveness of mitochondrial subpopulations to hyperglycemia and metabolic stress with the enhanced susceptibility of IFM in T1DM suggest that IFM could be the preferred target for drug treatments to prevent cardiomyopathy. The mitochondrial dysfunction in the hyperglycemic condition was assessed mainly in animal models. There is a lack of research aimed at learning the mechanisms of the development of cardiomyopathy in human T1DM patients. This research will be important to determine therapeutic goals acting against the emergence of T1DM. The results of animal studies are encouraging to test the above-mentioned substances in people with T1DM.

## Figures and Tables

**Table 1:** Changes in mitochondrial function based on various animal models.

Author	Year	Animal Model of T1DM	Changes in Mitochondrial Function
Ferreira et al. [11]	2013	rats	decrease in all respiratory complexes activity, except complex III increase in one of the uncoupling proteins of the IMM–UCP-3 decrease in the mitochondrial level of cytochrome c
Tocchetti et al. [12]	2015	guinea pigs	decrease in respiration, but only with complex II and IV substrates markedly lower ADP phosphorylation rates
Vazquez et al. [13]	2015	rats	decreased NADH coenzyme Q oxidoreductase and succinate-coenzyme Q oxidoreductase of complexes I and II, respectively
Vadvalkar et al. [14]	2017	Akita mice	impaired state 3 respiration with physiological pyruvate acetylation within mitochondrial pyruvate carrier 2
Pham et al. [15]	2014	rats	suppression of ATP synthesis capacity in oxygen-saturated states
Bugger et al. [16]	2009	Akita mice	decrease in state 3 respiration and ATP synthesis repression of OXPHOS subunits with reduced expression of OXPHOS subunit genes

**Abbreviations:** T1DM—type 1 diabetes mellitus, IMM—inner mitochondrial membrane, UCP-3—uncoupling protein-3, ADP—adenosine diphosphate, NADH—nicotinamide adenine dinucleotide hydride, ATP—adenosine triphosphate, OXPHOS—oxidative phosphorylation.

**Table 2:** Options of treatment of adverse changes induced by hyperglycemia in cardiac mitochondria in type 1 diabetes mellitus.

Author	Year	Country	Test Factor	Study Group	Effect of the Examined Factor on Mitochondria
Ye et al. [19]	2004	USA	Catalase	mice	reduction of MDA level and excessive production of ROS, prevention of morphologic damage to mitochondria
Semaming et al. [9]	2014	Thailand	Insulin and protocatechuic acid	rats	decrease in MDA level and ROS production, attenuation of mitochondrial depolarization and mitochondrial swelling, increase in anti-apoptotic BCL2 protein expression
Tocchetti et al. [12]	2015	USA	Insulin and palmitate	guinea pigs	restoration of proper cardiac redox balance (both substances), protection of mitochondrial respiration (only palmitate)
Da Silva et al. [32]	2015	Brazil	Insulin and swimming training	rats	correction of such pathologies as reduced [Ca(^2+^)]I transient, increased uncoupling protein-2 expression, increased Ca(^2+^) uptake (swimming training), further normalization of Ca^2+^ transient amplitude, NADPH oxidase-4 expression and carbonyl protein contents in left ventricular (LV) tissue (insulin), restoration of LV tissue superoxide dismutase and mitochondrial O_2_ consumption, H_2_O_2_ release and permeability transition pore (MPTP) opening in heart mitochondria (combined therapy)
Remor et al. [33]	2011	Brazil	Insulin	rats	protection against the hyperglycemia-induced inhibition of mitochondrial OXPHOS enzymes activities
Makino et al. [20]	2010	USA	O(^2^)(_-_) scavenger TEMPOL	mice	decrease in mitochondrial fragmentation, oxidative stress (significant decrease in oxidative stress marker 8-iso-PGF-2α) and the oxidized level of several proteins
Thapa et al. [25]	2015	USA	Mitofilin	Mice	preservation of ETC complexes I, III, IV, V activities, state 3 respiration, mitochondrial membrane potential, damage of cristae structure and decrease in the accumulation of lipid peroxidation by-products
Baseler et al. [36]	2013	USA	Mitochondria phospholipid hydroperoxide glutathione peroxidase 4	Mice	restoration of state 3 and state 4 respiration rates, preservation of mitochondrial respiratory chain proteins and ETC complex I, III, and IV activities, increase in ATP synthase activity, reversal of mitochondrial protein import dysfunction, decrease in H_2_O_2_ production and accumulation of lipid peroxidation by-products
Guo et al. [37]	2015	China	Aldehyde dehydrogenase 2	Mice	promoting the AMPK-dependent autophagy
Zhang et al. [40]	2016	China	Low-dose radiation at medium or high doses (25 or 50 mGy)	Mice	inhibition of cardiac P53 activation, suppression of the increased ratio of Bax to Bcl, decrease in the contents of such classic oxidative damage markers as 3-NT, 4-HNE and MDA, inhibition of ROS production, improvement of Akt activation and increase in Nrf2 function

**Abbreviations:** MDA—malondialdehyde, ROS—reactive oxygen species, NADPH—nicotinamide adenine dinucleotide phosphate hydride, OXPHOS—oxidative phosphorylation, 8-iso-PGF-2α—8-iso-prostaglandin F2α, ETC—electron transport chain, ATP—adenosine triphosphate, AMPK—AMP-dependent protein kinase, 3-NT—3-nitrotyrosine, 4-HNE—4-hydroxy-2-nonenal, Nrf2—nuclear factor erythroid 2-related factor 2.
